# Explore the feasibility of using spot‐scanning proton arc therapy for a synchrotron accelerator‐based proton therapy system – A simulation study

**DOI:** 10.1002/acm2.14526

**Published:** 2024-09-17

**Authors:** Xiaoda Cong, Gang Liu, Peilin Liu, Lewei Zhao, Shupeng Chen, Xiaoqiang Li, Jiajian Shen, Xuanfeng Ding

**Affiliations:** ^1^ Department of Radiation Oncology Corewell Health William Beaumont Hospital Royal Oak Michigan USA; ^2^ Cancer Center, Union Hospital, Tongji Medical College Huazhong University of Science and Technology Wuhan China; ^3^ Department of Radiation Oncology Stanford University California USA; ^4^ Department of Radiation Oncology Mayo Clinic Arizona USA

**Keywords:** intensity‐modulated proton therapy, spot scanning proton arc therapy, synchrotron‐accelerator

## Abstract

**Objective:**

The aim of this study was to evaluate the feasibility and plan quality of spot‐scanning proton arc therapy (SPArc) using a synchrotron‐accelerator‐based proton therapy system compared to intensity‐modulated proton therapy (IMPT).

**Approach:**

Five representative disease sites, including head and neck, lung, liver, brain chordoma, and prostate cancers, were retrospectively selected. Both IMPT and SPArc plans are generated with the HITACHI ProBEAT PBS system's minimum MU constraints and physics beam model. The SPArc plans are generated with 2.5° sampling frequency. The static delivery time was simulated based on the previously published synchrotron delivery sequence model, and the dynamic delivery time was simulated using a proton arc gantry mechanical model integrated with the synchrotron delivery sequence. Both dosimetric plan quality and delivery efficiency are evaluated.

**Main results:**

A superior plan quality is reached compared with the IMPT plans generated for the same disease site. However, a relatively prolonged static and dynamic delivery time post new challenge, as static time increased by 49.22% and dynamic time 59.10% on average.

**Significance:**

This study presents the first simulation results of delivering the SPArc plans using a synchrotron‐accelerated proton therapy system. The result shows its feasibility and limitations, which could guide future development.

## 1 INTRODUCTION

Since the introduction of Spot‐scanning Proton Arc (SPArc) therapy in 2016,^1^ it has been drawing significant interest among the radiation oncology community because of superior dosimetric plan quality,^2^ efficient treatment delivery workflow,^3^ and capability of better LET modulation^4^ compared to the conventional intensity‐modulated proton therapy (IMPT). Recent investigations revealed that various disease sites or clinical indications would benefit via the SPArc technique, including prostate,^5^ breast,^6^ brain,^7^ head and neck,^8^ lung,^9^ and liver^10^ cancer patients. These exciting findings drove our community to develop this technology for clinical implementation further.^11^ In 2018, through an academic‐industrial partnership, the prototype DynamicARC system was successfully installed and tested in a clinical environment, which demonstrated the feasibility of this new treatment modality.^2^

However, all the previous research and developments are based on the cyclotron or synchrocyclotron accelerator systems using a degrader as an energy layer selection system. None of the studies has been done to explore the feasibility of using a synchrotron‐ accelerator‐based proton therapy system (PTS). One of the main concerns is that the effective dose rate is much lower than the other two peers, including slow extraction, pro‐ longed cycling time, and limited maximum charges per spill.^12^ As a result, previous studies found that it takes much longer to irradiate an IMPT plan by a synchrotron accelerator compared to the cyclotron or synchrocyclotron accelerator PTS,^13^ let alone a more complicated SPArc plan with many more energy layers, which could trigger more beam cycling time during the irradiation. Thus, developing SPArc therapy using a synchrotron‐accelerator‐based PTS for routine clinical practice seems to be an untouchable ceiling. Such understanding and thinking also barred or discouraged the investigators from developing the SPArc therapy using a synchrotron‐accelerator‐based PTS or treatment planning system (TPS). To overcome such barriers, there is an immediate need to address this challenge—Is a synchrotron accelerator system feasible for any proton arc therapy? If so, what is the expected treatment delivery time with current synchrotron accelerator and beamline technology?

To answer these fundamental questions, as the first step, our community needs to understand the current limitations of using a synchrotron‐accelerator‐based PTS for SPArc therapy, such as dosimetric plan quality and treatment delivery time, before drawing any conclusion on the feasibility. Thus, we performed the first quantitative investigation by generating a series of standard SPArc plans for various disease sites and clinical indications using the physics beam model from a synchrotron‐accelerator‐based PTS to (1) explore if there are any similar dosimetric improvements compared to the IMPT using synchrotron‐accelerator‐based PTS compared to the previous findings using other accelerators, and (2) simulate its treatment delivery time and efficiency based on a synchrotron delivery sequence model^14^ and integrated with the proton arc gantry mechanical model,^3^ so that our community can have strong reference data to assess the feasibility of routine clinical implementation.

## 2 METHOD AND MATERIALS

### 2.1 Patient selections and SPArc plan generations

#### 2.1.1 Physics beam model

A physics beam model from a HITACHI (Hitachi, Ltd, Tokyo, Japan) ProBEAT PBS system, Mayo Clinic Arizona was configured in RayStation (RaySearch Laboratories AB, Stockholm, Sweden) TPS version 6.0. The ProBEAT system utilizes a synchrotron ac‐ celerator, with 97 discrete energies ranging from 71.3 to 228.8 MeV and discrete spot scanning. More details about this synchrotron accelerator‐based PTS can be found in a prior publication.^14^

#### 2.1.2 Patient selection

**FIGURE 1:**
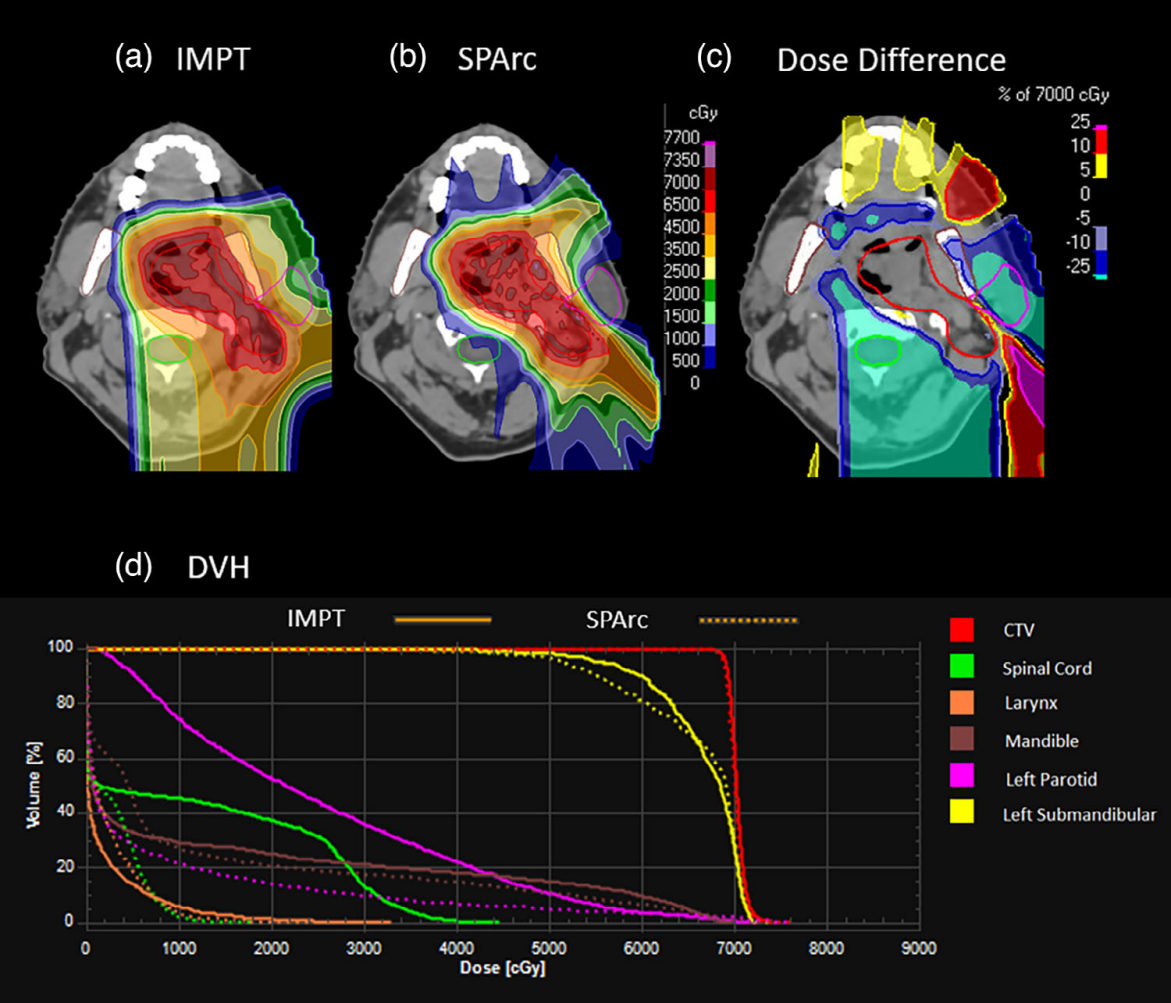
The plan quality and DVH comparison between IMPT and SPArc plan of the unilateral head and neck case. (a) IMPT; (b) SPArc; (c) dose difference between IMPT and SPArc nominal plan; (d) DVH shows the dosimetric difference between IMPT(solid line) and SPArc(dash line).

Five representative cases were selected in this study, including head and neck, lung, liver, brain chordoma, and prostate cancers from Corewell Health, William Beaumont University Hospital, Proton Therapy Center. Detailed information about the selected cases is included in Table 1, and the HN is unilateral with detailed disease site information included in the supplement document (Figure 1s). The Institutional Review Board approved the study (2017‐455).

**TABLE 1:** Disease and treatment plan information of the selected case.

				IMPT		SPArc		
Tumor site	CTV (cc)	Dose (cGy)	Fractions	No. of fields	No. of energy layers	No. of control points	No. of energy layers	Arc range (deg)
H&N	129.07	7000	35	3(G70,T0)^a^ (G75,T340) (G180,T0)	123	176	176	Partial arc (0–180)
Lung	93.88	6000	30	3(G0,T180) (G160,T180) (G186,T180)	115	128	128	Partial arc (180–360)
Liver	175.93	6750	15	2(G130,T180) (G186,T180)	108	139	139	Partial arc (180–360)
Chordoma	34.15	5040	28	3(G0,T0) (G130,T180) (G230,T180)	91	214	214	Full arc (0–360)
Prostate	41.42	3800	5	2(G90,T0) (G270,T0)	34	152	152	Full arc (0–360)

^a^ G: Gantry angle; T: Table angle.

#### 2.1.3 SPArc and IMPT plan generation

For each case, a robustly optimized IMPT plan was generated in RayStation TPS using this HITACHI physics beam model. The new IMPT plan used the same beam angles, setup uncertainty (2–5 mm depending on the disease site), and range uncertainty (3.5%) as the original clinical plan. The IMPT plans served as the baseline for the dosimetry comparison. Detailed information about the IMPT plan is included in Table 1. The SPArc plan was generated using an in‐house developed optimization algorithm implemented in the RayStation TPS (version 6.0) via scripting. A brief summary of the SPArc plan generation for this study is included here, and more details can be found in the previous literature.1 First, a coarse sampling of multiple fields robustly optimized IMPT plan was generated. The coarsely sampled IMPT plan at 20° sampling frequency is the starting point of the SPArc plan. Next, an iterative process was applied to gradually increase the number of control points until it reached the desired sampling frequency, for example, 2.5°.

The SPArc algorithm takes care of redistributing the energy layers to each control point. To maintain the delivery efficiency of SPArc plan, the algorithm also performs iterative energy layer filtration or selection in which the energy layers or the whole control point with low weighting were removed if the plan quality was not degraded. In this study, a single arc was used for SPArc plan, keeping one energy per control point. The arc trajectories were either full arc or partial arc based on the patient's geometry and target locations. Detailed information about the SPArc plan is included in Table 1. The IMPT and SPArc plans are robustly optimized with the same setup uncertainty (2–5 mm depending on disease site) and range uncertainty (3.5%) under 20 worst‐case scenarios.

### 2.2 Dosimetry comparisons between IMPT and SPArc plans

The plan quality was compared between IMPT and SPArc planning groups, including dose‐volume histograms (DVHs) of the target and organs at risk (OARs). The conformity index1 (CI) is defined as:

(1) $$\begin{array}{ccc} & & CI\;=\\ & & \frac{\mathrm{patient}\;\mathrm{body}\;\mathrm{volume}\;\mathrm{receiving}\;95\%\;\mathrm{prescription}\;\mathrm{dose}\;}{\mathrm{CTV}} \end{array}$$

DVH metrics were used to assess the plan quality, and the IMPT and SPArc planning groups were compared including D99% for CTV. Maximum doses for OARs: spinal cord, brainstem, cochlea, and optic nerves and mean dose for OARs: esophagus, liver, kidney, larynx, mandible, parotid, submandibular, heart, lung, bladder, and rectum. and V5Gy and V20Gy for lungs.

In addition to nominal plan evaluation in target coverage and OARs sparing, the integral dose (ID) is also evaluated which is defined as:

(2) $$ID\left(Gy*L\right)=D\left(Gy\right)*V\left(L\right)$$

where *D* is the mean dose delivered to total volume V.^15^

### 2.3 Treatment delivery time comparisons between IMPT and SPArc plans

To assess the treatment delivery efficiency, two kinds of beam delivery times (a) static beam delivery time (BDT*_s_*) and (b) dynamic beam delivery time (BDT*_d_*) were simulated and compared between IMPT and SPArc plans.

BDTs simulation was performed based on the previous publication on the Hitachi ProBEAT system single energy extraction (SEE) mechanism. The BDT*_s_* only includes irradiation time, such as spot drill time, spot switching time, and cycling time, without considering the gantry rotation time between beam angles.

The BDTs model for a HITACHI ProBEAT SEE system used in this study is summarized below^14^:

(3) $$\begin{array}{ccc} \;\;T_{BDT} & = & T_{LSw}+T_{SSp}+T_{SSw}\\ & = & \underset{i=1}{\sum^{N_{layer}}}\;t_{LSw}^{i}\times \left(1+m_{EX}^{i}+m_{MU}^{i}\right)+\underset{i=1}{\sum^{N_{layer}}}\;\frac{MU_{i}}{{\dot{P}}_{i}}\\ & & +\;\underset{i=1}{\sum^{N_{layer}}}\;\underset{j=1}{\sum^{N_{spot_{i}}}}\;\frac{\left|x_{i,j+1}-x_{i,j}\right|}{V_{x}^{i}}+\frac{\left|y_{i,j+1}-y_{i,j}\right|}{V_{y}^{i}}+t_{SMag}^{i,j} \end{array}$$

where the total beam delivery time is composed of three components: energy layer switch time (*T_LSw_*), spot spill time (*T_SSp_*), and the spot switch time (*T_SSw_*). These three components were further expanded into per individual layer and per spot with machine‐specific parameters in Equation (1), where $\;N_{layer}$ is the total number of energy layers in the beam, $\;N_{spot_{i}}$ is the total number of spots in a given energy layer i. The term $t_{LSW}^{i}$ is the energy layer switch time for a given energy, and the term $1+m_{EX}^{i}+m_{MU}^{i}$ means that the number of acceleration cycles for given energy may be more than one, where $m_{EX}^{i}$ and $m_{MU\;}^{i}$ stands for the number of additional acceleration cycle required in layer i when there are not sufficient protons in the synchrotron and when the time to deliver the MU for that layer is longer than the maximum time that the synchrotron can hold the protons stably, respectively. Of note, $m_{EX}^{i}$ and $m_{MU}^{i}$ are not needed for most of the clinical plans unless a big amount of MU or spot numbers are needed for a given layer. The spot spill time equals the required MU for a given layer ($MU_{i}$) divided by the proton spill rate $\overset{.}{\underset{i}{P}}$ (MU/s). The per‐spot switch time includes the scanning magnet verification time ($t_{SMag}^{i,j}$) and the scanning magnet steering time equals the distance between the current spot and the next spot ($|x_{i,j+1}-\;x_{i,j}|$ and $|y_{i,j+1}-\;y_{i,j}|$) divided by the energy‐specific effective scanning magnet speeds, $V_{x}^{i}$ and $V_{y}^{i}$.

All the machine‐specific parameters used for HITACHI ProBEAT system are based on the publication from Shen et al. ^14^ In summary, the ELST is about 2 s, the scanning magnet verification time is about 2 ms, the proton spill rate is about 8−10 MU/s, the total protons from one acceleration are about 2 nC, the maximum time to hold these charges in the accelerator is about 8 s, and the scanning speeds are 5−7 m/s (*V_x_*) and 17−22 m/s (*V_y_*).

BDT*_d_* simulation was performed, taking into account the gantry mechanical limitations and rotation time such as maximum gantry acceleration or deceleration speed and maximum gantry velocity.^3^ More specifically, the dynamic arc system controller calculates the BDT*_s_* (Equation 1) in each individual control point and connects the gantry rotation between the adjacent control point without violating the mechanical constraints such as maximum gantry speed (6 deg/s), maximum gantry acceleration (0.6 deg/s2), and deceleration speed (0.5 deg/s2).^3^ The IMPT simulation of BDT_d_ calculation assumes the gantry stops while irradiating the proton spots and energy layers. Then, it calculates the gantry rotation time between the static IMPT field without considering the data transfer time between OIS and PTS, room switching time, beam request, and preparation time. In other words, IMPT BDT*_d_* simulates an ideal situation, such as an auto step‐and‐shoot IMPT plan.

## 3 RESULTS

### 3.1 Plan quality comparison between SPArc and IMPT plans

The results of IMPT and SPArc plan quality comparisons are shown in Table 2. SPArc plans show significantly reduced maximum dose to the spinal cord in the liver, HN, and lung cases and improved target dose conformity for all five cases.

**TABLE 2:** Dosimetric comparison between IMPT and SPArc nominal plans for different disease sites.

Structure	Value	IMPT	SPArc
HN case			
CTV	D99% (Gy)	68.35	68.26
Spinal cord	Max (Gy)	38.59	11.76
Larynx	Mean (Gy)	2.07	2.54
Mandible	Max (Gy)	67.75	69.95
Left parotid	Mean (Gy)	25.04	8.26
Left submandibular	Mean (Gy)	66.46	65.71
Left brachial plexus	Mean (Gy)	14.07	11.71
Oral cavity	Mean (Gy)	10.81	11.61
Oral cavity	Max (Gy)	62.04	58.55
Brain stem	Max (Gy)	11.20	2.20
CI		4.73	2.51
Lung case			
CTV	D99% (Gy)	56.89	57.53
Esophagus	Mean (Gy)	3.18	2.97
Heart	Mean (Gy)	1.73	1.60
Lungs‐CTV	Mean (Gy)	8.35	7.23
Lungs‐CTV	V5Gy (%)	22.59	28.30
Lungs‐CTV	V20Gy (%)	16.74	14.08
Spinal cord	Max (Gy)	13.98	4.72
CI		3.46	2.88
Liver case			
CTV	D99% (Gy)	66.64	66.02
Esophagus	Mean (Gy)	7.18	7.73
Right kidney	Mean (Gy)	0.01	0.24
Liver	Mean (Gy)	14.20	11.44
Spinal cord	Max (Gy)	25.85	8.46
CI		2.38	2.11
Chordoma case			
CTV	D99% (Gy)	50.02	50.38
Brainstem	Max (Gy)	52.34	50.49
Left cochlea	Max (Gy)	28.91	11.02
Right cochlea	Max (Gy)	45.64	19.89
Left optic nerve	Max (Gy)	52.15	50.50
Right optic nerve	Max (Gy)	51.66	49.69
Optic chiasm	Max (Gy)	52.02	49.79
CI		5.76	3.87
Prostate case			
CTV	D99% (Gy)	36.96	36.67
Bladder	Mean (Gy)	3.97	2.99
Rectum	Mean (Gy)	3.58	2.08
CI		2.96	2.55

Figure 1 shows the example of HN cancer and DVH comparison between SPArc and IMPT. With similar target coverage (D99:SPArc: 68.26 Gy vs. IMPT: 68.35 Gy), the SPArc plan shows a significant reduction in the maximum dose of spinal cord compared to IMPT (Max:SPArc: 11.76 Gy vs. IMPT: 38.59 Gy) as well as a major decrease to the mean dose to left parotid (Mean:SPArc: 8.26 Gy vs. IMPT: 25.04 Gy) as shown in Table 2. The CI improves for SPArc plan(SPArc: 2.51 vs. IMPT: 4.73).

**FIGURE 2:**
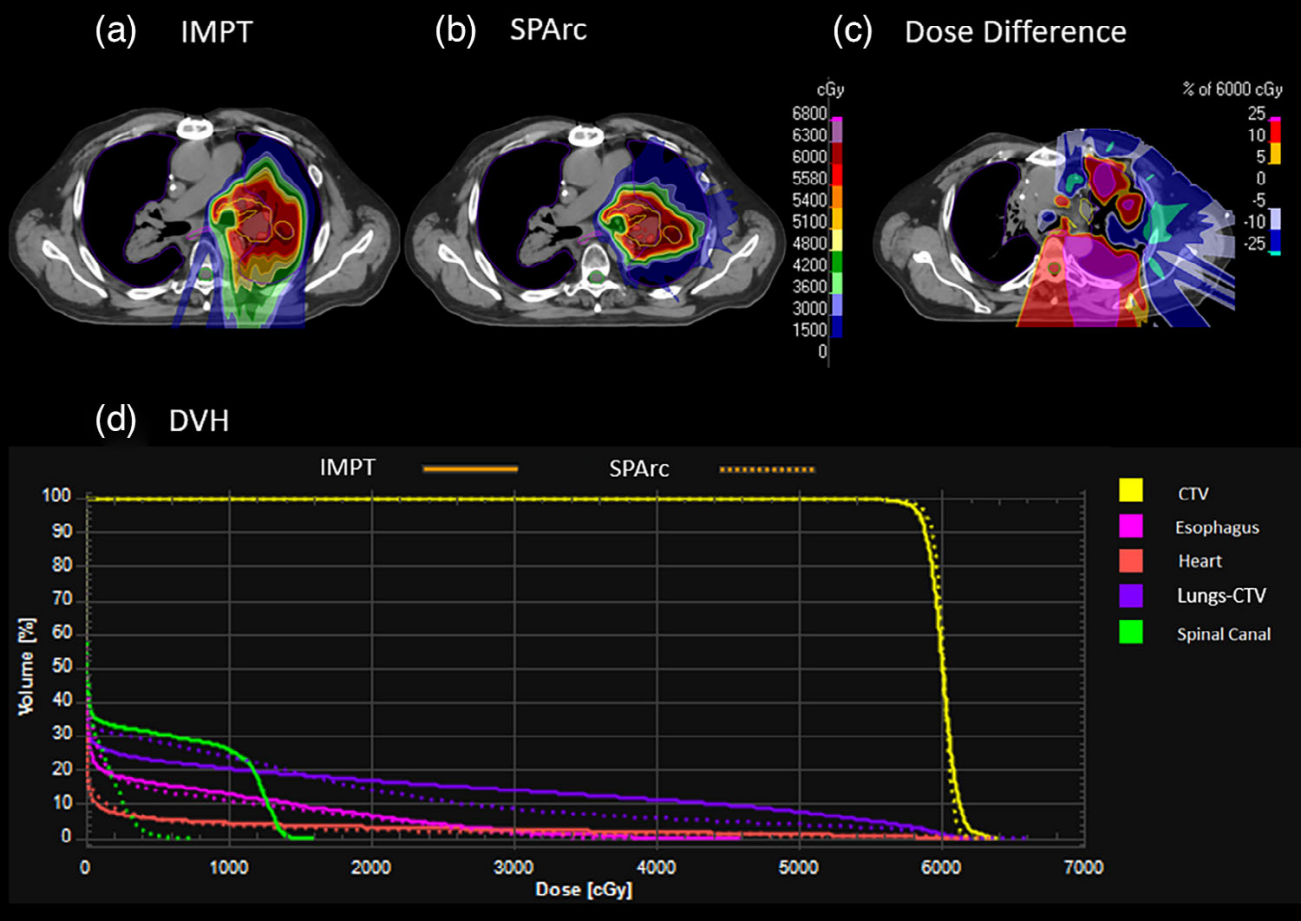
Dose distribution and DVH for lung case IMPT and SPArc plan. (a) IMPT; (b) SPArc; (c) dose difference between IMPT and SPArc nominal plan; (d) DVH shows the dosimetric difference between IMPT(solid line) and SPArc(dash line).

Figure 2 shows the example of lung cancer and DVH comparison between SPArc and IMPT. With a similar target coverage (D99: SPArc: 57.53 Gy vs. IMPT: 56.89 Gy), SPArc was able to reduce the maximum dose to the spinal cord from 13.98  to 4.72 Gy compared to IMPT plan. Additionally, SPArc was able to improve CI (SPArc: 2.88 vs. IMPT: 3.46). It was found that SPArc could also reduce V20Gy of the healthy lung (Lungs‐CTV) from 16.74% to 14.08%, though V5Gy increased from 22.59% to 28.30% compared to the IMPT.

**FIGURE 3:**
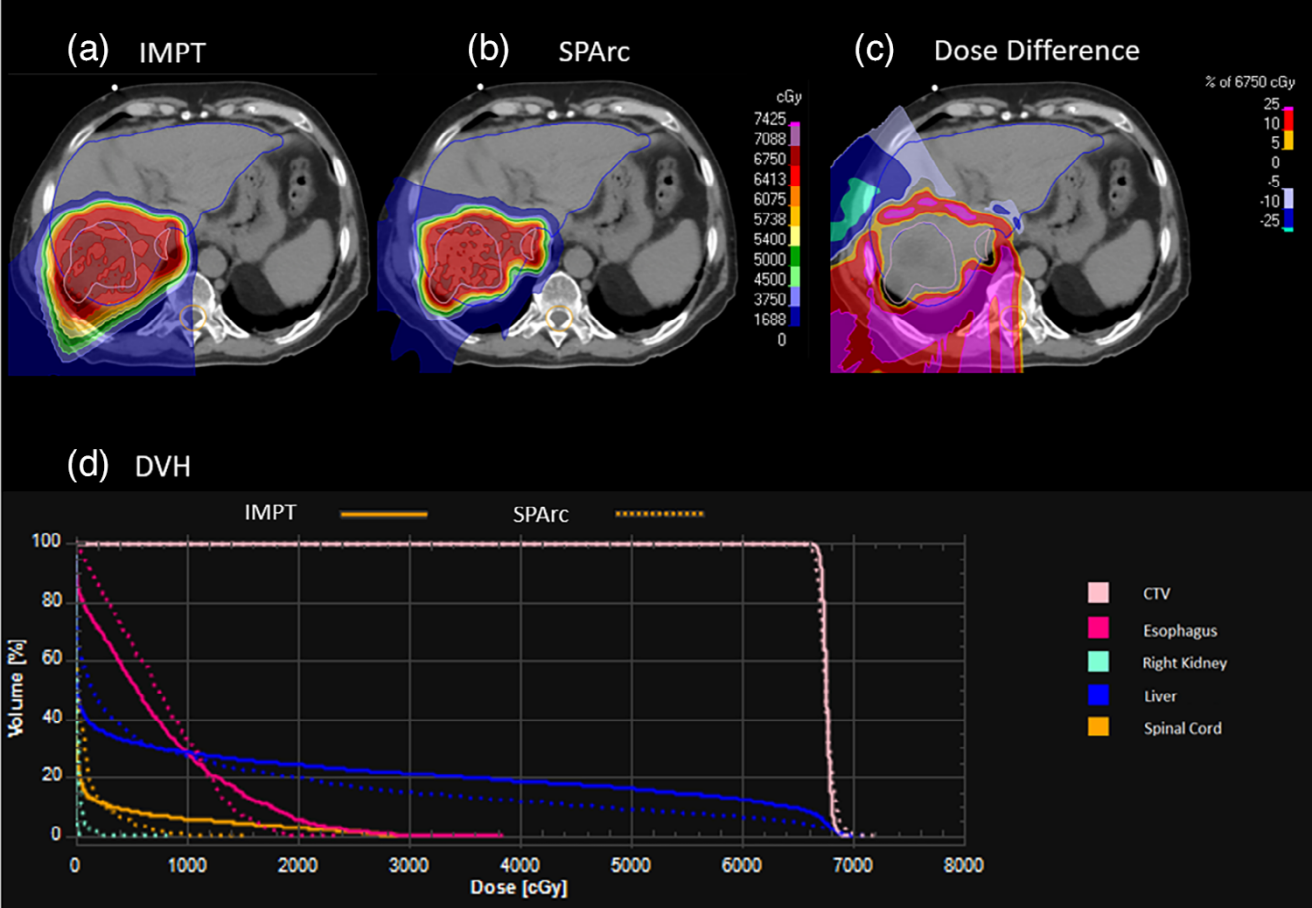
Dose distribution and DVH for liver case IMPT and SPArc plan. (a) IMPT; (b) SPArc; (c) dose difference between IMPT and SPArc nominal plan; (d) DVH shows the dosimetric difference between IMPT (solid line) and SPArc (dash line).

Figure 3 shows the example of liver cancer and DVH comparison between SPArc and IMPT. With a similar target coverage (D99:SPArc: 66.02 Gy vs. IMPT: 66.64 Gy), the maximum dose to the spinal cord could be reduced using SPArc (Max: SPArc:8.46 Gy vs. IMPT:25.85 Gy). The mean dose to the esophagus was slightly increased (Mean:SPArc: 7.73 Gy vs. IMPT: 7.18 Gy). The CI is also improved by adapting SPArc(SPArc: 2.11 vs. IMPT: 2.38).

**FIGURE 4:**
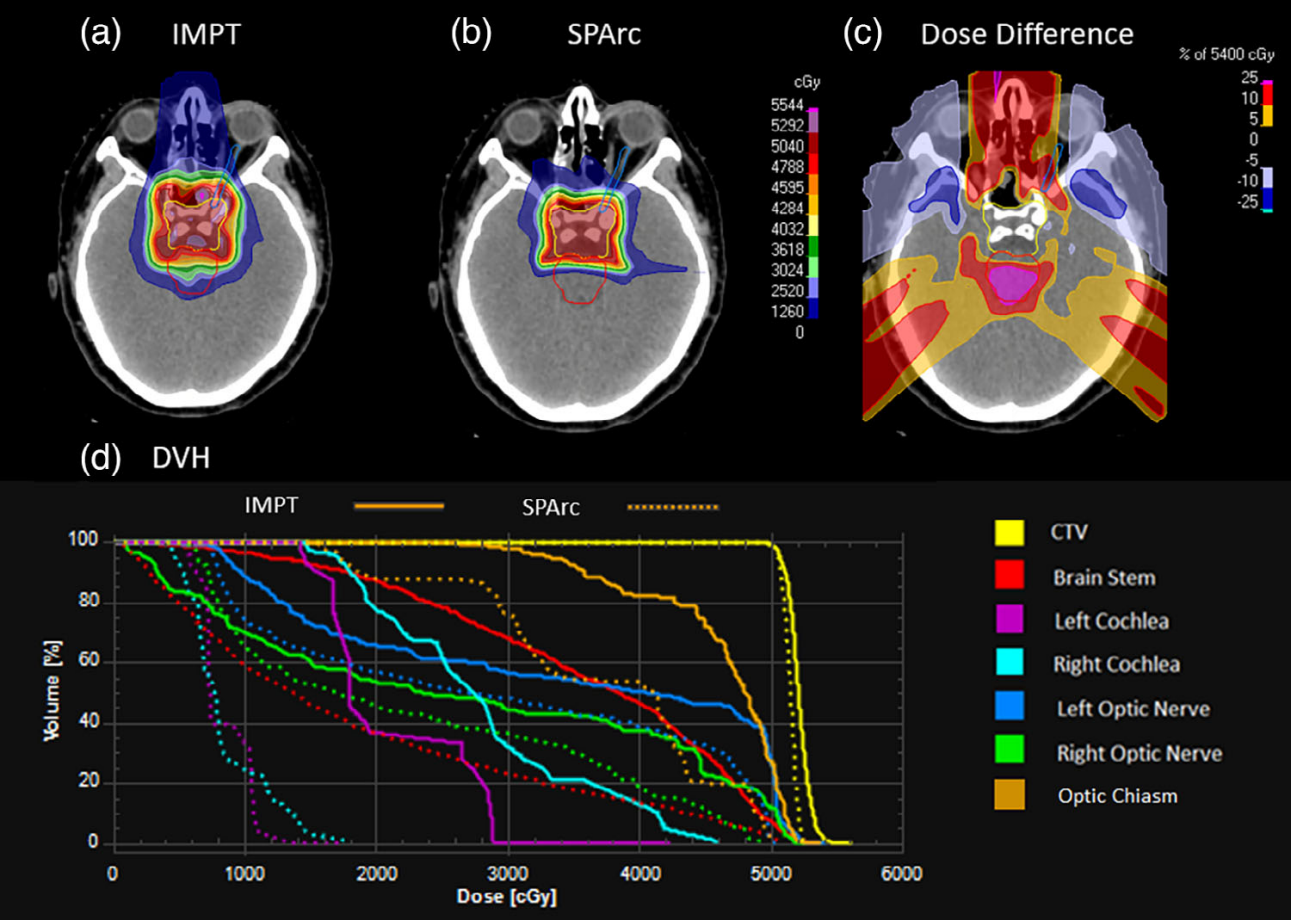
Dose distribution and DVH for brain chordoma cancer IMPT and SPArc plans. (a) IMPT; (b) SPArc; (c) dose difference between IMPT and SPArc nominal plan; (d) DVH shows the dosimetric difference between IMPT (solid line) and SPArc (dash line).

Figure 4 shows the example of brain chordoma cancer and DVH comparison between SPArc and IMPT. Similar target coverage was found for the SPArc plan and IMPT plan (D99:SPArc: 50.38 Gy vs. IMPT: 50.02 Gy). For the brain stem, the maximum dose was slightly reduced in the SPArc plan(Max:SPArc: 50.49 Gy vs. IMPT: 52.34 Gy). SPArc significantly reduces the maximum dose to the cochlea compared to IMPT (Max: SPArc: 11.02 Gy vs. IMPT: 28.91 Gy for left cochlea and Max:SPArc: 19.89 Gy vs. IMPT: 45.64 Gy for right cochlea). There was also a slightly improved maximum dose to the optic nerve(Max:SPArc: 50.50 Gy vs. IMPT: 52.15 Gy for left optic nerve and Max:SPArc: 49.69 Gy vs. IMPT: 51.66 Gy for the right optic nerve). The CI was also improved using SPArc technique (SPArc: 3.87 vs. IMPT: 5.76).

**FIGURE 5:**
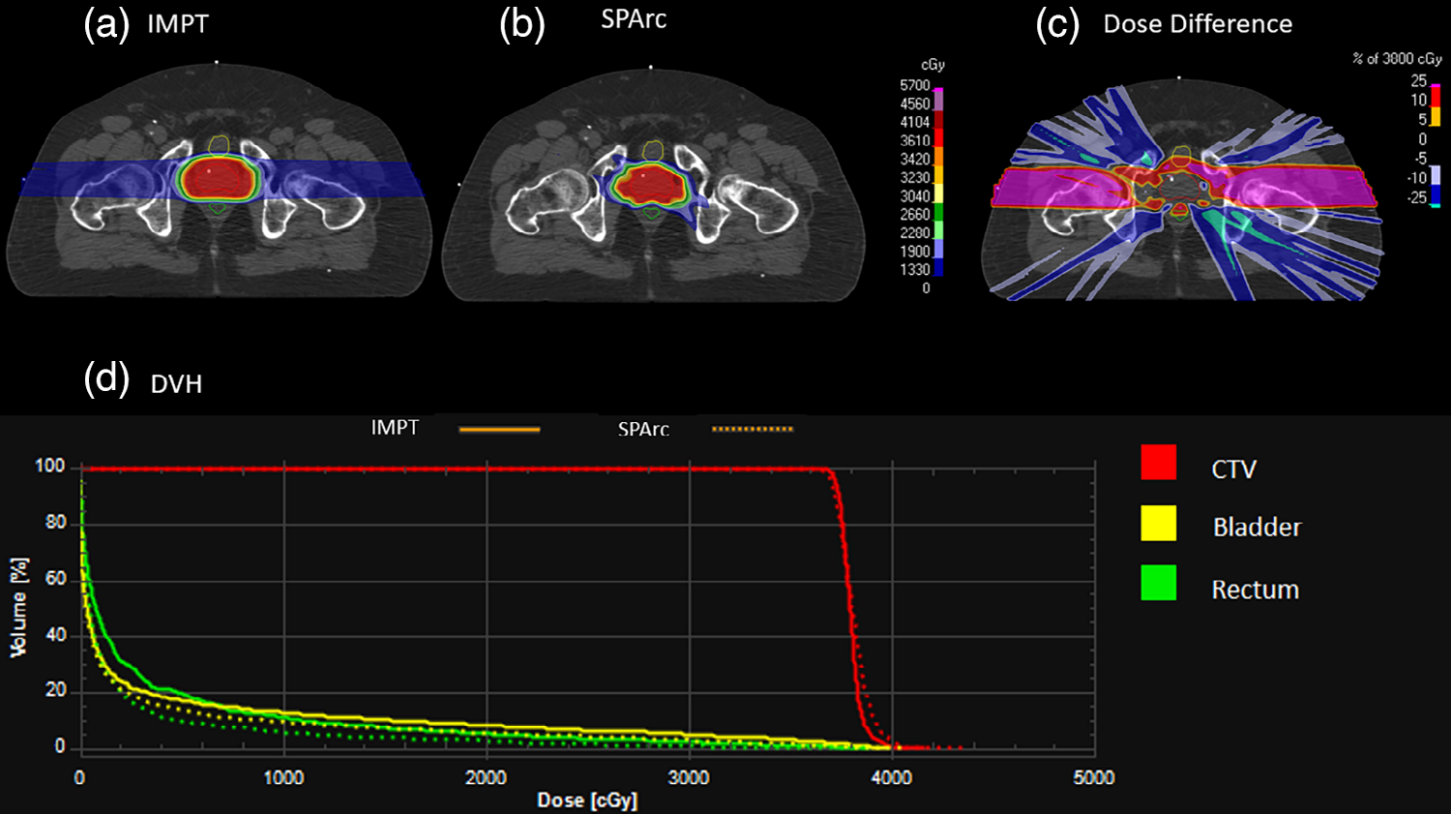
Dose distribution and DVH for prostate cancer IMPT and SPArc plans. (a) IMPT; (b) SPArc; (c) dose difference between IMPT and SPArc nominal plan; (d) DVH shows the dosimetric difference between IMPT (solid line) and SPArc (dash line).

Figure 5 shows the example of prostate cancer and DVH comparison between SPArc and IMPT. Similar target coverage was found for the SPArc plan and IMPT plan (D99:SPArc: 36.67 Gy vs. IMPT: 36.96 Gy). For bladder, the mean dose was slightly reduced in the SPArc plan (Mean:SPArc: 2.99 Gy vs. IMPT: 3.97 Gy). The mean dose to rectum is also reduced in SPArc plan (Mean:SPArc: 2.08 Gy vs. IMPT: 3.58 Gy). The CI was also improved using SPArc technique (SPArc: 2.55 vs. IMPT: 2.96).

For ID, as shown in Table 3, SPArc plans exhibit a 1.90%–24.56% lower ID compared to their corresponding IMPT plans.

**TABLE 3:** Integral dose comparison between IMPT and SPArc plans for different disease sites.

Disease site	Integral dose (Gy*L)		Integral dose difference
	IMPT	SPArc	(IMPT‐SPArc)/IMPT
HN	140.85	106.25	24.56%
Liver	97.67	80.18	17.91%
Lung	84.80	83.19	1.90%
Chordoma	41.43	32.77	20.90%
Prostate	26.77	24.54	8.33%

### 3.2 Beam delivery efficiency comparison between SPArc and IMPT plans

Table 4 shows the estimated beam delivery time per fraction for both IMPT and SPArc plans for each disease case. Overall, the SPArc plans showed a prolonged delivery time compared to IMPT in the given five cases, and the relative efficiency of SPArc became worse compared to IMPT, especially when the target volume is relatively small.

**TABLE 4:** IMPT and SPArc time efficiency on different volume of disease site.

	IMPT		SPArc		BDT_s_ increment	BDT_d_ increment
Disease site	BDT_s_ (s)	BDT_d_ (s)	BDT_s_ (s)	BDT_d_ (s)	(SPArc‐IMPT)	(SPArc‐IMPT)
HN	274.21	319.85	375.38	426.86	36.90%	33.46%
Lung	269.26	323.79	317.73	395.48	18.00%	22.14%
Liver	331.97	340.53	402.17	442.12	21.15%	29.83%
Chordoma	182.13	273.41	420.14	544.89	130.68%	99.29%
Prostate	95.83	130.47	133.58	274.97	39.39%	110.75%
Average	230.68	277.61	329.80	416.86	42.97%	50.16%

For the base of the skull chordoma case, the BDT*_s_* and BDT*_d_* were increased by (SPArc: 420.14 s vs. IMPT: 182.13 s) 238.01 s and (SPArc: 544.89 s vs. IMPT: 273.41 s)271.48s, respectively, using SPArc compared with IMPT. For the HN case, the SPArc plan's BDT*_s_* and BDT*_d_* were increased by (SPArc: 375.38 s vs. IMPT: 274.21 s) 101.17 s and (SPArc: 426.86 s vs. IMPT: 319.85 s) 107.01 s, respectively, compared with IMPT. For lung

case, the BDT_s_ and BDT_d_ were increased by (SPArc: 317.73 s vs. IMPT: 269.26 s) 48.47 s and (SPArc: 395.48 s vs. IMPT: 323.79 s) 271.48 s, respectively, using SPArc compared with IMPT. For the liver case, the BDT_s_ and BDT_d_ were increased by (SPArc: 402.17 s vs. IMPT: 331.97 s) 70.2 s and (SPArc: 442.12 s vs. IMPT 340.53 s) 101.59 s, respectively, using SPArc compared with IMPT. For the prostate case, the BDT_s_ and BDT_d_ were increased by (SPArc: 133.58 s vs. IMPT: 95.83 s) 37.75 s and (SPArc: 274.97 s vs. IMPT 130.47 s) 144.50 s, respectively.

### 3.3 Robustness optimization comparison between IMPT and SPArc plans

In Table 5, the robustness evaluation is conducted for all five disease sites, including measurements of mean dose, D95, and V95, expressed as percentages normalized to the prescription dose. The result shows equivalent robust performance in SPArc plans compared to IMPT plans. Perturbed DVHs were also included in the Figure S2–S6.

**TABLE 5:** Worst‐case scenarios evaluation of different disease sites.

Disease site		IMPT	SPArc
HN	Mean dose (%)	100.40 (100.21∼100.53)	100.14 (100.01∼100.31)
	D95 (%)	98.46 (97.96∼98.70)	98.09 (97.83∼98.41)
	V95 (%)	99.20 (98.80∼99.30)	99.30 (99.30∼100.00)
Lung	Mean dose (%)	99.92 (98.93∼101.38)	100.23 (99.92∼100.52)
	D95 (%)	96.50 (94.80∼97.80)	98.40 (97.72∼98.68)
	V95 (%)	97.30 (94.30∼98.80)	98.90 (98.30∼99.30)
Liver	Mean dose (%)	100.18 (99.93∼100.41)	99.96 (99.75∼100.19)
	D95 (%)	99.20 (98.93∼99.41)	98.24 (97.91∼98.61)
	V95 (%)	100.00 (99.30∼100.00)	99.20 (98.80∼99.30)
Chordoma	Mean dose (%)	103.35 (102.32∼104.40)	102.98 (102.50∼103.37)
	D95 (%)	100.04 (98.29∼101.33)	100.28 (99.52∼101.27)
	V95 (%)	99.80 (99.30∼100.00)	99.30 (99.30∼100.00)
Prostate	Mean dose (%)	99.89 (99.32∼100.47)	100.11 (99.45∼100.68)
	D95 (%)	97.84 (97.11∼98.53)	96.37 (94.68∼97.39)
	V95 (%)	98.50 (97.30∼99.30)	97.30 (94.30∼99.30)

## 4 DISCUSSION

The study reported the first SPArc therapy simulation study using a synchrotron‐accelerator‐based PTS. The treatment delivery time (BDT*_s_* and BDT*_d_*) was simulated using a validated and published beam delivery time model.^3^, ^14^ The preliminary result showed that SPArc can provide a superior dosimetric plan quality over the conventional IMPT over various disease sites, such as target dose conformity, which is similar to the previous publication using cyclotron or synchrocyclotron accelerator PTS. Such a technique could potentially improve treatment outcomes and prognosis with the synchrotron‐accelerator based PTS.

However, due to the machine characteristics of the synchrotron accelerator, the study found that the treatment delivery time could be significantly prolonged due to the numerous cycling periods based on the SEE mechanism, which could make the clinical implementation of SPArc technology more challenging via synchrotron‐accelerator based PTS.

There are several limitations in this study. First, the BDT is based on the SEE mechanism. Thus, the total number of energy layers remains the bottleneck of using a synchrotron accelerator to deliver SPArc plan. Recently, multi energy extraction (MEE) technique has been gradually implemented in the clinical PTS, which could improve both IMPT and SPArc treatment delivery efficiency.^16^ The BDT we saw with the SEE synchrotron accelerator could differ from that with the MEE synchrotron accelerator. Second, the study assumed a full‐circle gantry for planning and BDT estimations. Since a half‐circle gantry is also used at some centers, the quantitative results in this work may not be directly applicable to these systems, as they may require additional couch/chair rotation to finish a full‐arc SPArc treatment.

The findings from this study also lead to some opportunities further to develop the software and hardware of PTS and TPS. For example, developing a new cycling technique^17^ could significantly reduce the treatment irradiation time. The introduction of the fast line scanning technique could solve the delivery time challenges for the disease sites with a large target volume.^18^ Furthermore, a new treatment plan optimization algorithm can be developed to reduce the energy layer number, leading to fewer cycling triggers without losing significant plan quality. A similar concept introduced previously using energy layer sparsity or reduction for cyclotron or synchrocyclotron accelerator could also benefit the synchrotron accelerator system.^19^

Of note, this planning study is preliminary work to test feasibility. We did not manually select arc trajectory to avoid beams passing through the couch edge or organs with day‐to‐day anatomical changes (e.g., rectum, bladder) because the current arc trajectory is based on inverse optimization and the objective functions have been applied to minimize the dose in these structures. However, it is possible to manually select the arc trajectory to avoid beam entrance. In this supplemental document, we replanned the prostate cancer case incorporating a manual avoidance structure for arc trajectory placement. The new plan shows similar quality to the full inverse optimized arc plan. Detailed dosimetric comparison results for this new plan (SPArc avoid OAR) are included in the supplemental document (Figure S7 and Table S1).

## 5 CONCLUSION

SPArc plans can provide a superior dosimetric plan quality compared with conventional IMPT plans via synchrotron‐accelerator based PTS. However, the study confirms the prolonged treatment delivery time compared to conventional clinical IMPT. This first quantitative information could help the subsequent development of SPArc technique for future efficient clinical implementation with a synchrotron‐accelerator based PTS.

## AUTHOR CONTRIBUTIONS

The authors confirm contribution to the article as follows: study conception and design: X. Cong, X Ding, J. Shen; data collection: X. Cong, L. Zhao, S. Chen, X. Li, J. Shen; analysis and interpretation of results: X. Cong, G. Liu, P. Liu, X. Ding, J. Shen, draft manuscript preparation: X. Cong, X. Ding, J. Shen. All authors reviewed the results and approved the final version of the manuscript.

## CONFLICT OF INTEREST STATEMENT

X. Ding and X. Li hold patents related to particle arc therapy, which has been licensed to IBA.

## Supporting information



Supporting information
