# Supplementary material for: Explore the feasibility of using spot‐scanning proton arc therapy for a synchrotron accelerator‐based proton therapy system – A simulation study
Source: J Appl Clin Med Phys. 2024 Sep 17;26(1):e14526. doi: 10.1002/acm2.14526 (PMC11713393; doi:10.1002/acm2.14526)
Supplement: Supplementary file 1 — Supporting information [file ACM2-26-e14526-s001.docx]

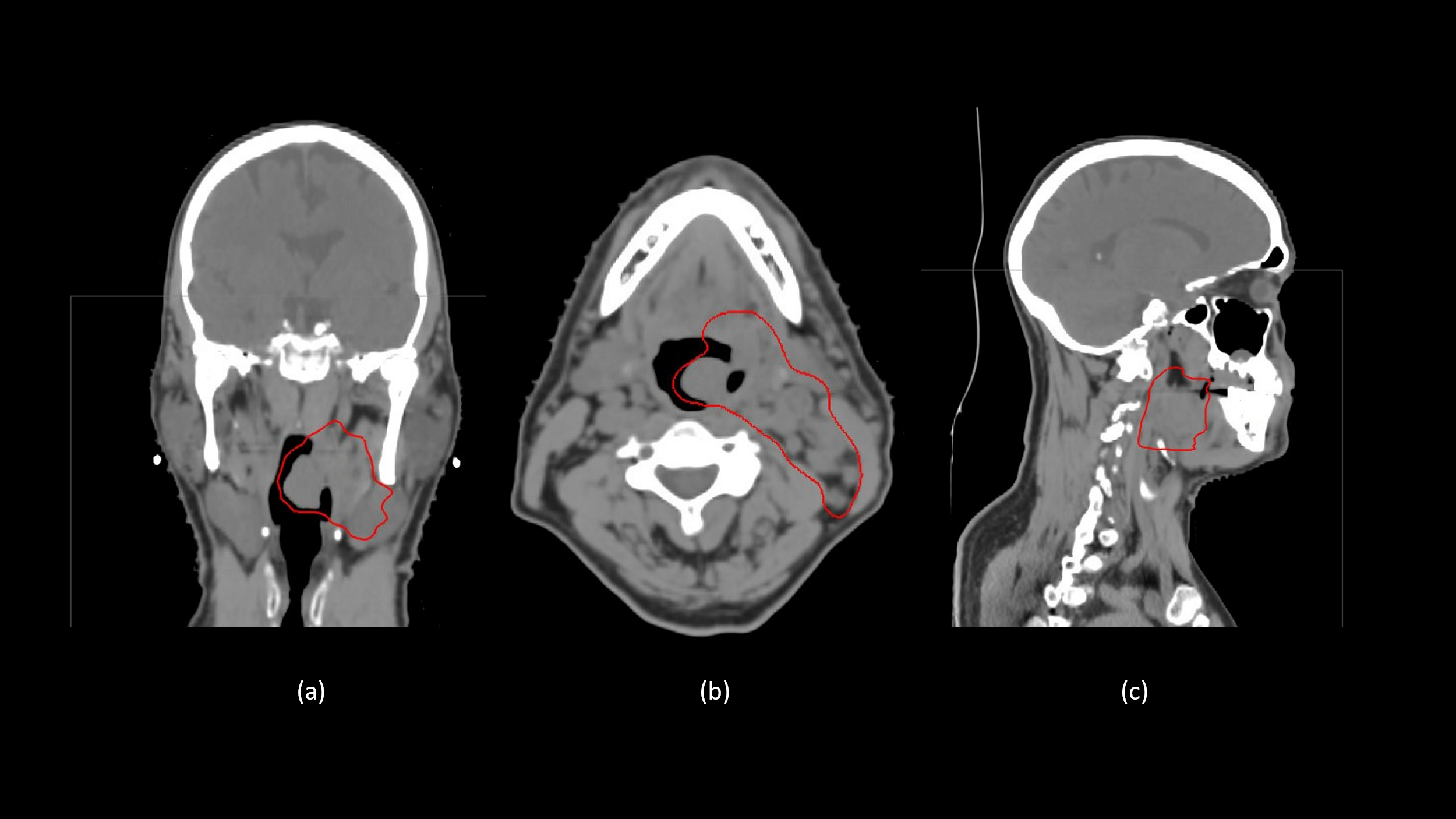


**Figure 1s** Disease site information about HN cancer. (a)is for coronal, (b) is for transversal, (c) is for sagittal.


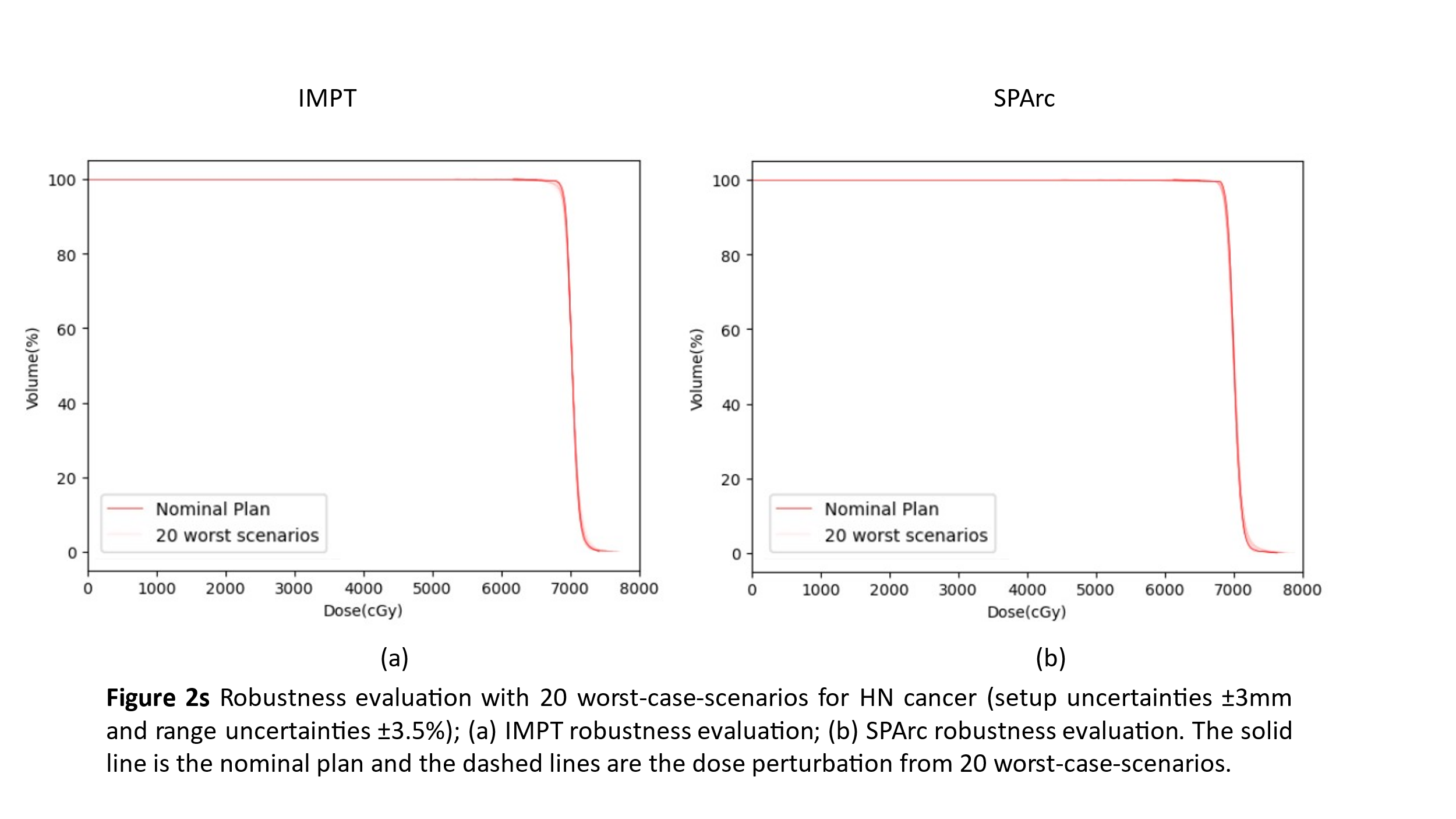


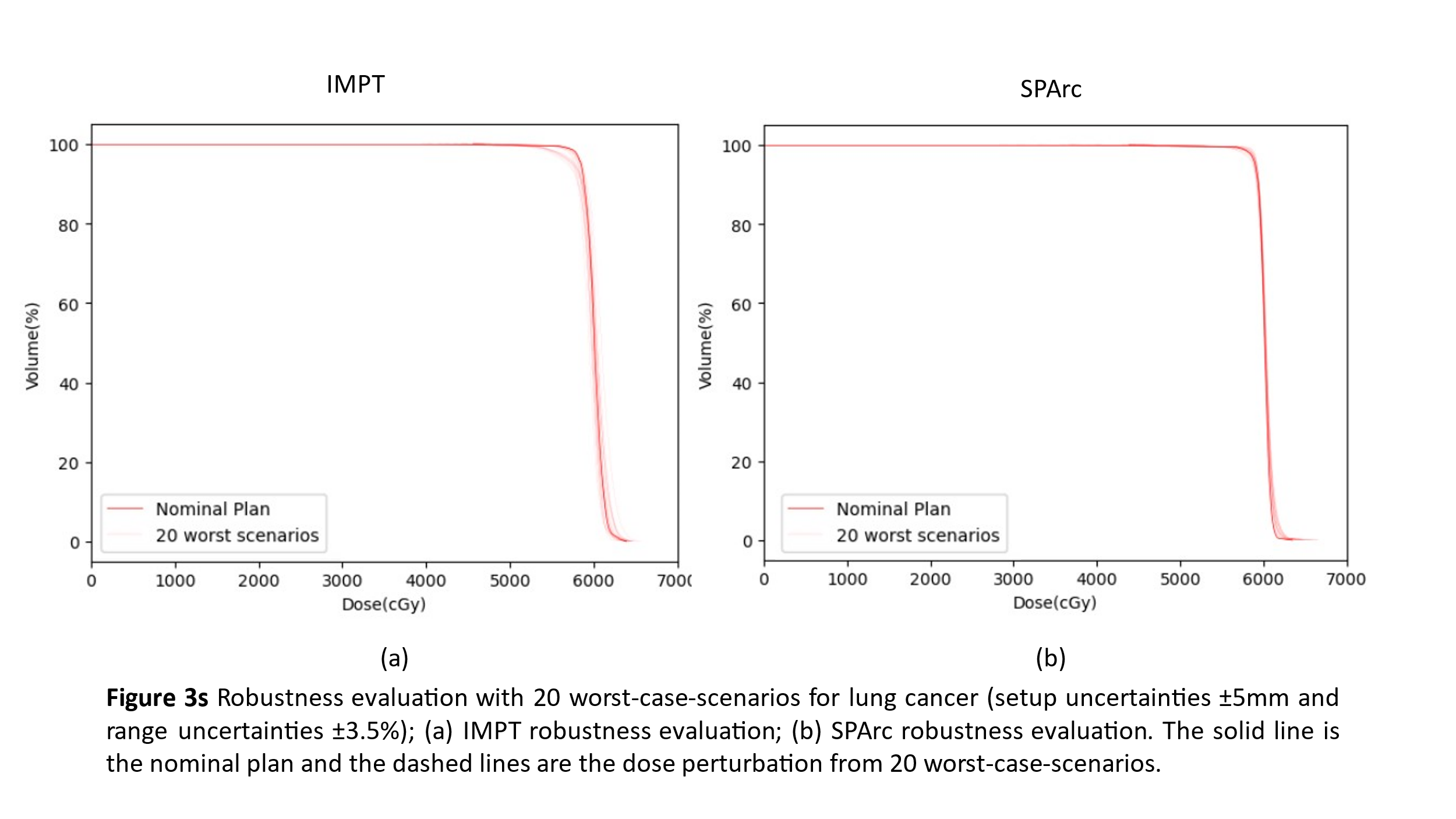

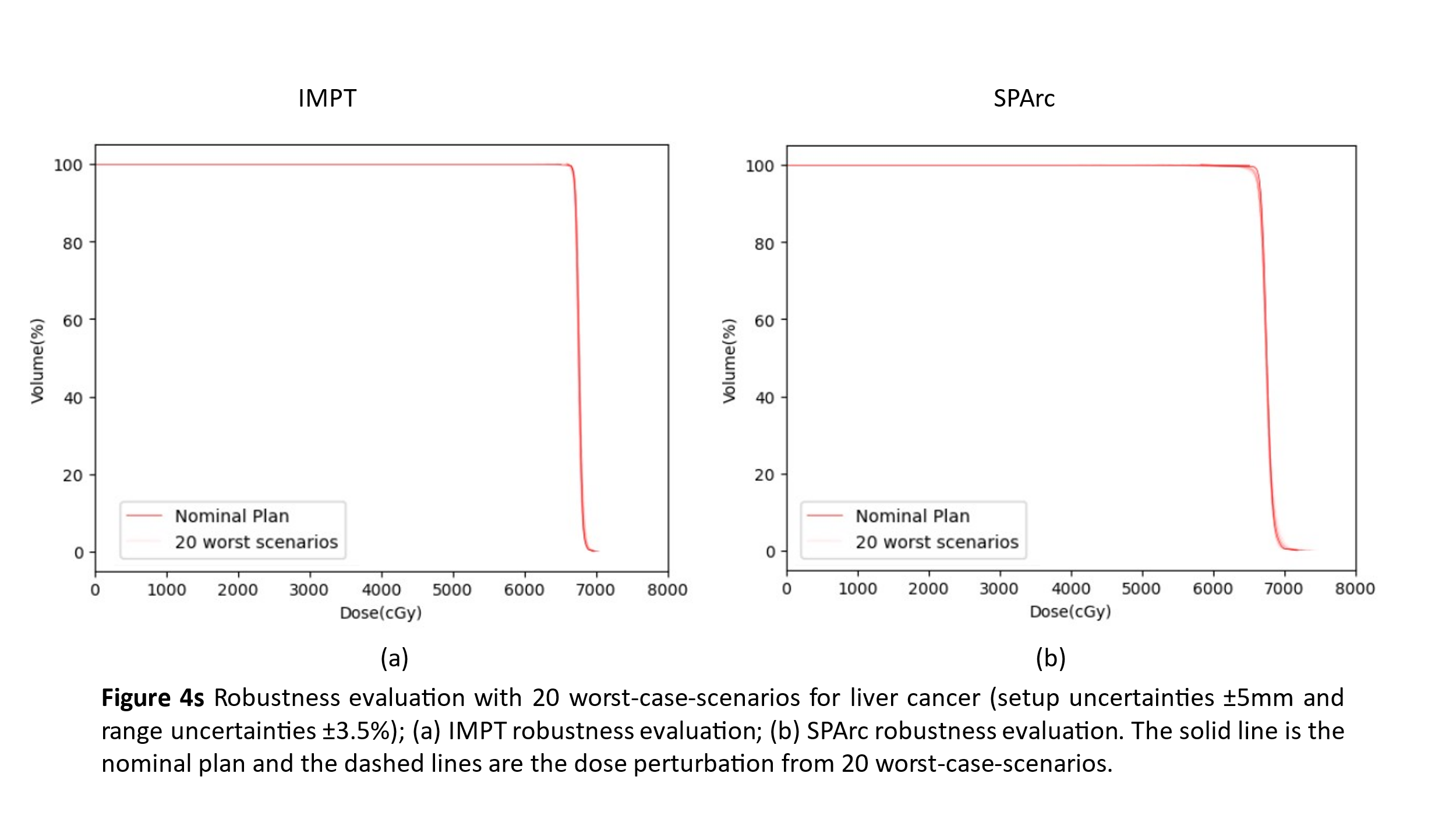


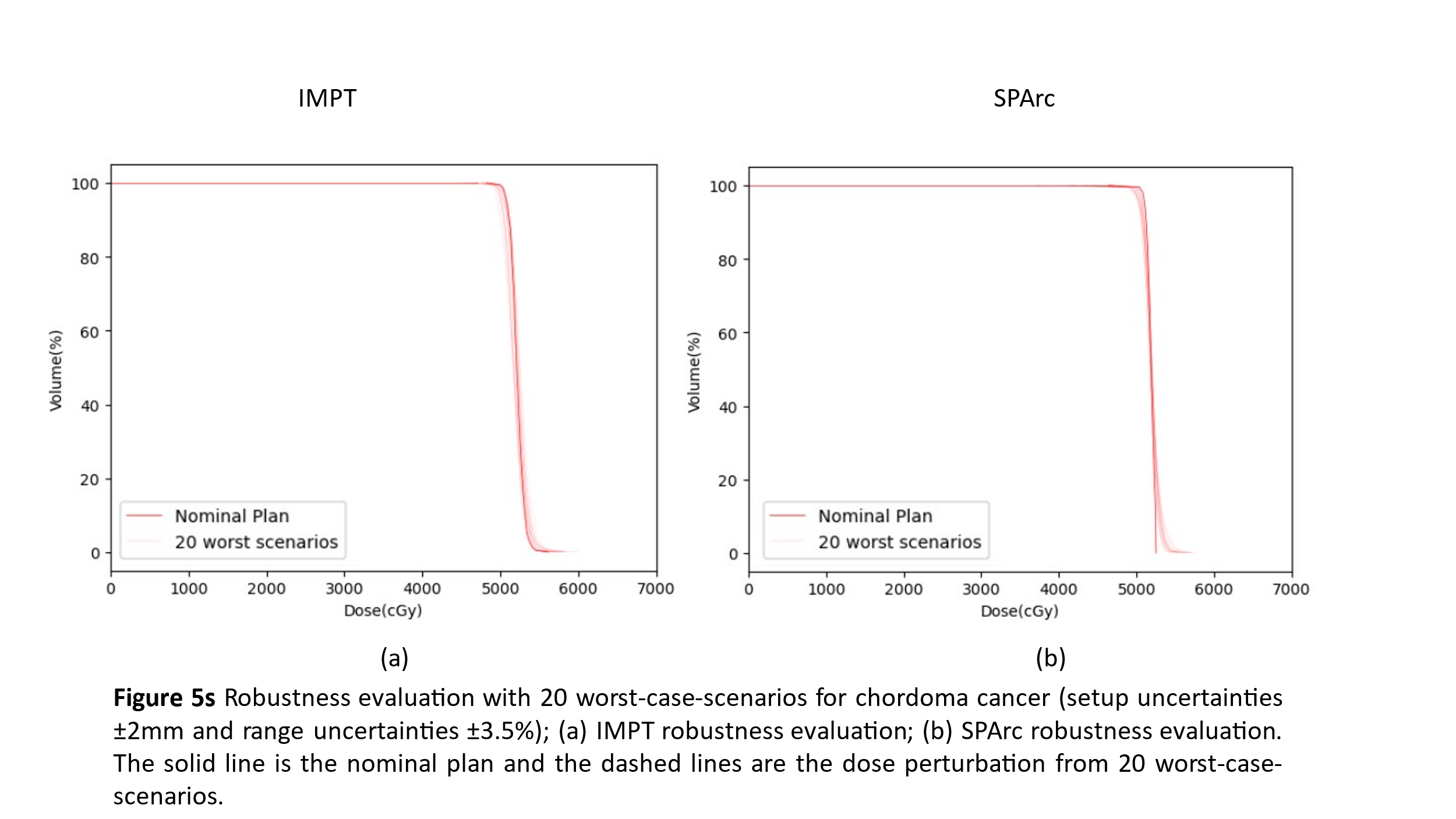


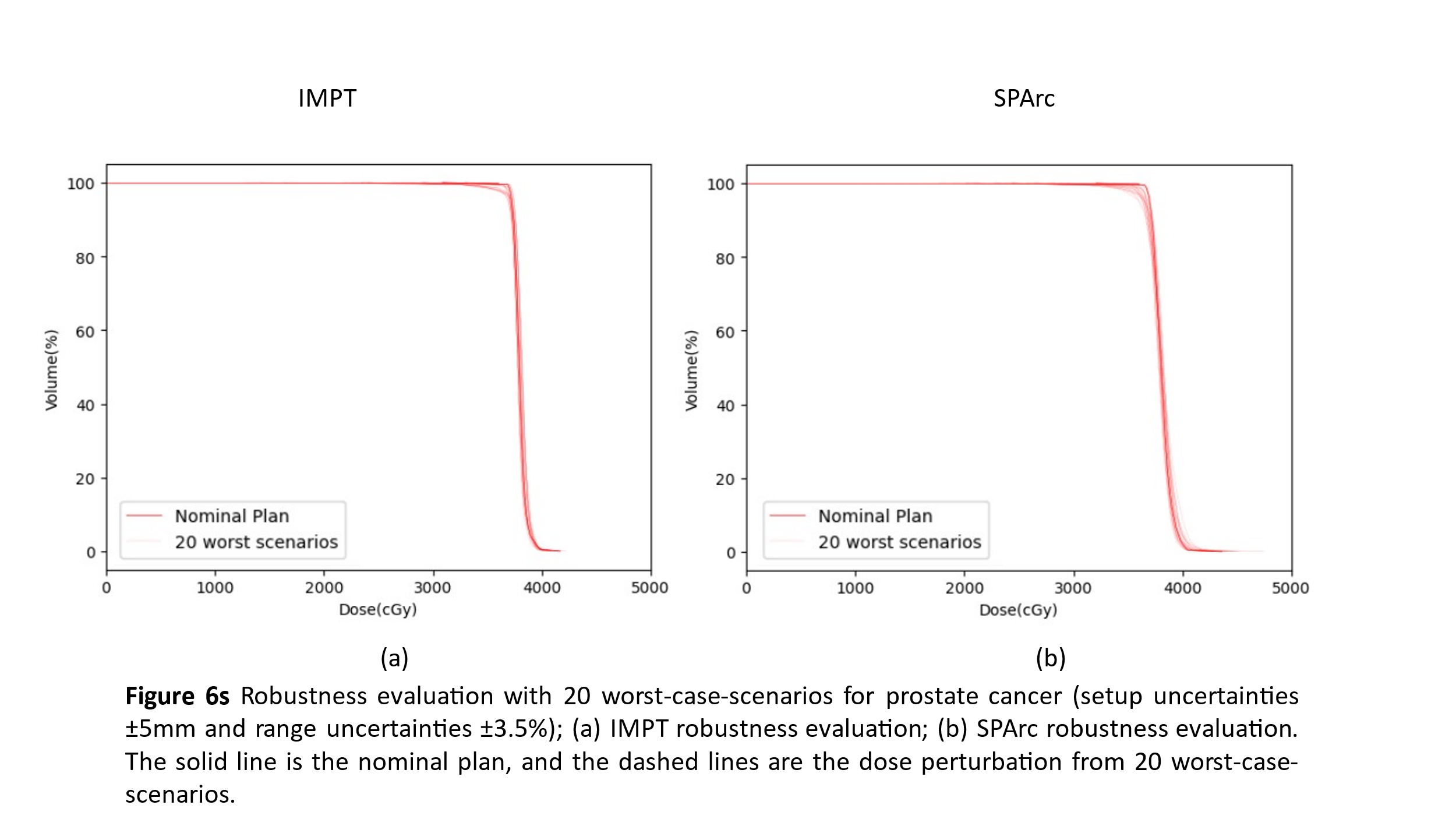


**Table 1s** Prostate case with table corner and OAR avoidance

| Structure | Value | IMPT | SPArc | SPArc(avoid OAR) |
| --- | --- | --- | --- | --- |
| CTV | D99%(Gy) | 36.96 | 36.67 | 36.73 |
| Bladder | Mean(Gy) | 3.97 | 2.99 | 3.05 |
| Rectum | Mean(Gy) | 3.58 | 2.08 | 2.08 |
| CI |  | 2.96 | 2.55 | 2.28 |


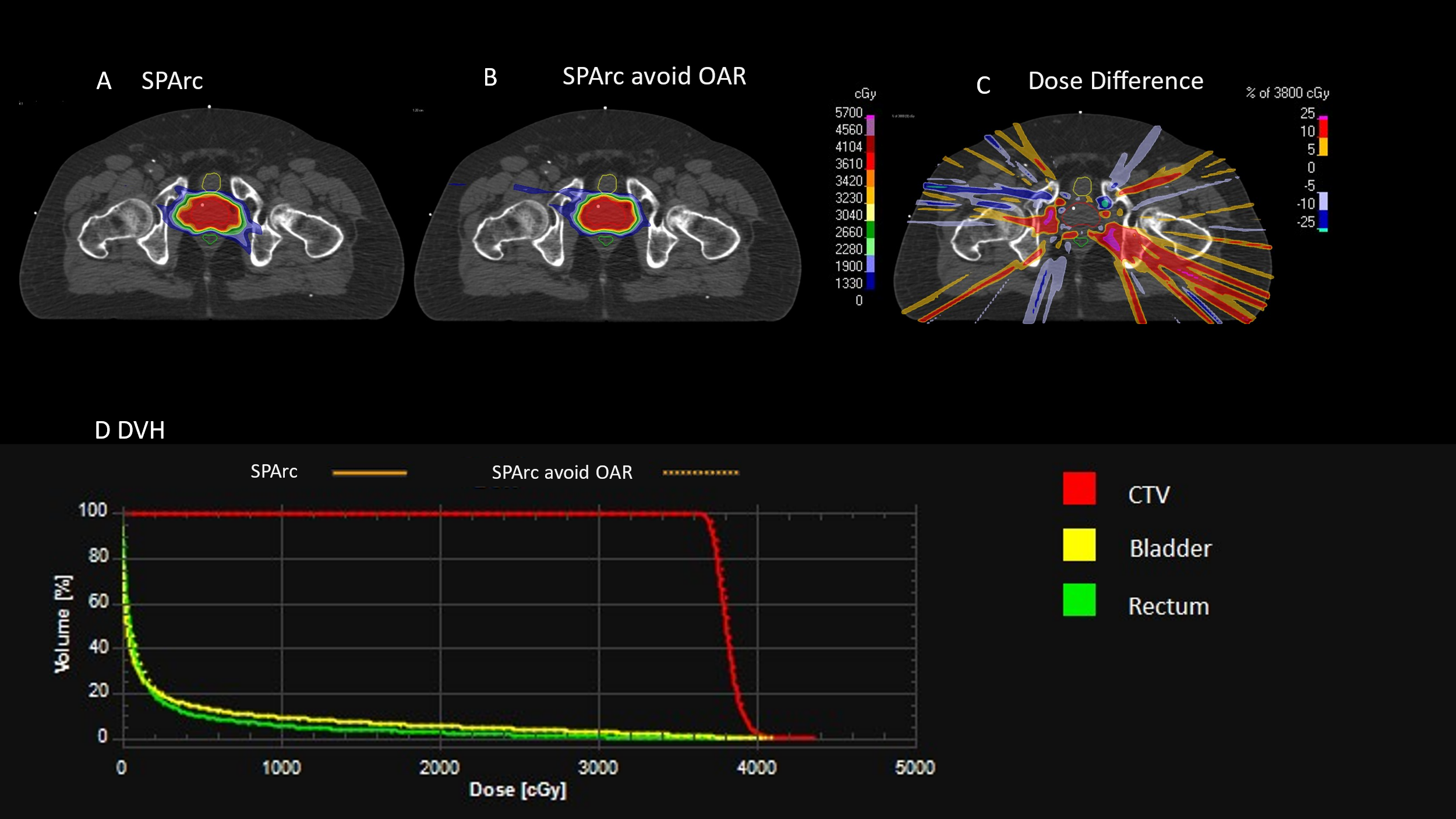


**Figure 7s** The plan quality and DVH comparison between IMPT and SPArc plan of prostate. (A) IMPT ;( B) SPArc; (C) dose difference between IMPT and SPArc plan; (D)DVH shows the dosimetric difference between IMPT(solid line) and SPArc(dash line).
